# Supplementary material for: COVID-19: Physical Activity and Quality of Life in a Sample of Swiss School Children during and after the First Stay-at-Home
Source: Int J Environ Res Public Health. 2022 Feb 16;19(4):2231. doi: 10.3390/ijerph19042231 (PMC8871913; doi:10.3390/ijerph19042231)
Supplement: Supplementary file 1 [file ijerph-19-02231-s001.zip › ijerph-1574200-supplementary.pdf]

Supplementary files for the manuscript

COVID-19: Physical activity and quality of life of Swiss school children during and after stay-at-home

Valentin Benzing[1], Patrice Gaillard[1], David Scheidegger[1], Alain Dössegger[2], Claudio R. Nigg[1], Mirko Schmidt[1]

[1]Institute of Sport Science, University of Bern, Bern, Switzerland
[2]Swiss Federal Institute of Sport SFISM, Magglingen, Switzerland

Correspondence concerning this article should be addressed to Valentin Benzing
Valentin Benzing, University of Bern, Bremgartenstrasse 145, 3012 Bern, Switzerland
E-mail: valentin.benzing@ispw.unibe.ch (VB)
Tel: 0041-31-631-45-48

Table S1. Comparison of background characteristics between children participating at both measurement points (*n* = 36) and those participating at the first measurement point only (*n* = 21).

	**Children participating at both measurement points** (*n* = 36)	**Children participating at the first but not second measurement point** (*n* = 21)	*P*
	Mean (*SD*)	*Mean* (*SD*)	
Age [years]	10.42 (1.11)	10.48 (1.78)	.891
Socioeconomic status [0-13]	9.56 (1.73)	8.14 (2.15)	.009 *
Height [cm]	146.09 (10.68)	135.40 (29.98)	.173
Weight [kg]	37.53 (8.39)	35.94 (10.26)	.501
BMI [kg/m^2]	17.23 (2.51)	18.04 (3.78)	.397
	n (%)	*n* (%)	
Overweight	4 (11.1%)	3 (14.3%)	.480
Sex [female]	18 (50%)	16 (23.8%)	.052
Swiss citizenship	36 (100%)	21 (100%)	
Swiss citizenship parents	34 (94.4%)	20 (95.2%)	.461
School grade (1st/ 3rd/ 4th/ 5th/ 6th)	0 (0%)/ 11 (30.6%)/ 6 (16.6%)/ 13 (36.1%)/ 6 (16.6%)	3 (14.3%)/ 3 (14.3%)/ 2 (9.5%)/ 9 (42.9%)/ 4 (19.0%)	.123
Membership in sports club	32 (88.9%)	13 (65.0%)	.031 *
Living conditions			
Residential area (urban, suburban, rural)	2 (5.6%)/ 8 (22.2)/ 26 (72.2)	1 (4.8%)/ 0 (0%)/ 20 (95.2%)	.062
Access to garden	35 (97.2%)	20 (95.2%)	.695
Nearby playground	32 (88.9%)	19 (90.5%)	.851
Pedestrian friendly neighborhood	33 (91.7%)	17 (81.0%)	.311

Note. SD = standard deviation; BMI = body mass index

Significant differences between the two groups are indicated by an asterisk (p < .05).*

Figure S1. Correlation between BMI and change in total score of HRQoL (r = -.334, p = .013) including overweight and normal weight children.

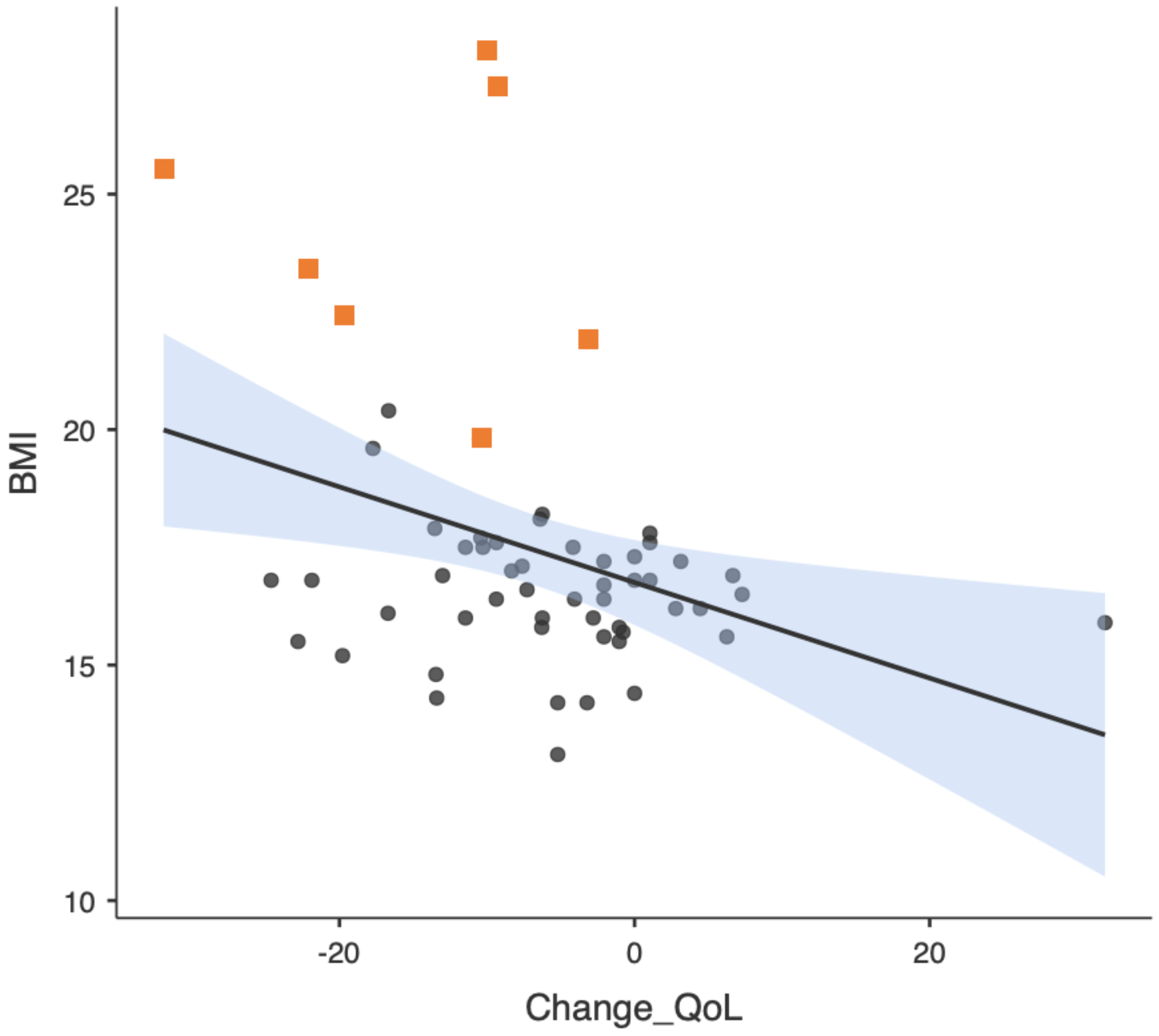

Note. The data depicted shows the mean value of imputed data sets for each participant. Change_QoL = total score of KINDL[R]. Orange-colored rectangles = overweight children. Black-colored dots = normal weight children.

Figure S2.

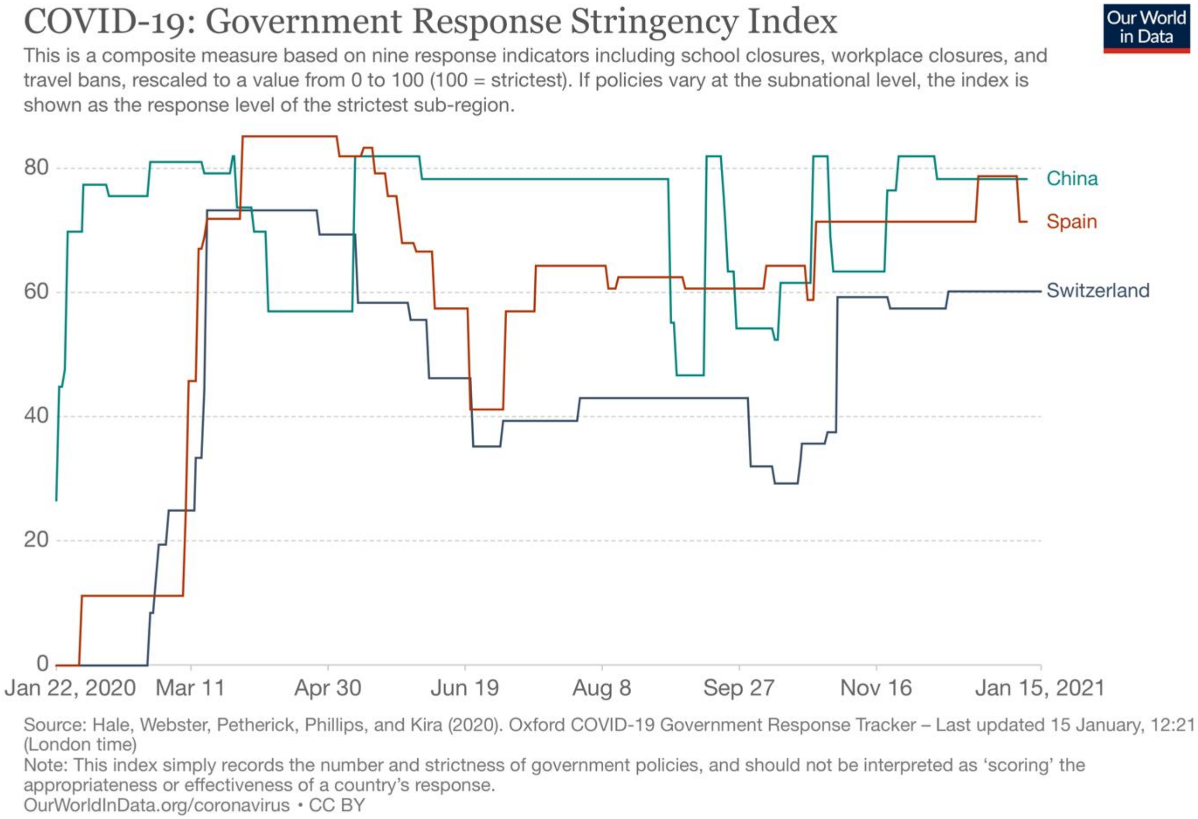

Source: https://ourworldindata.org/grapher/covid-stringency-index

Figure S3.

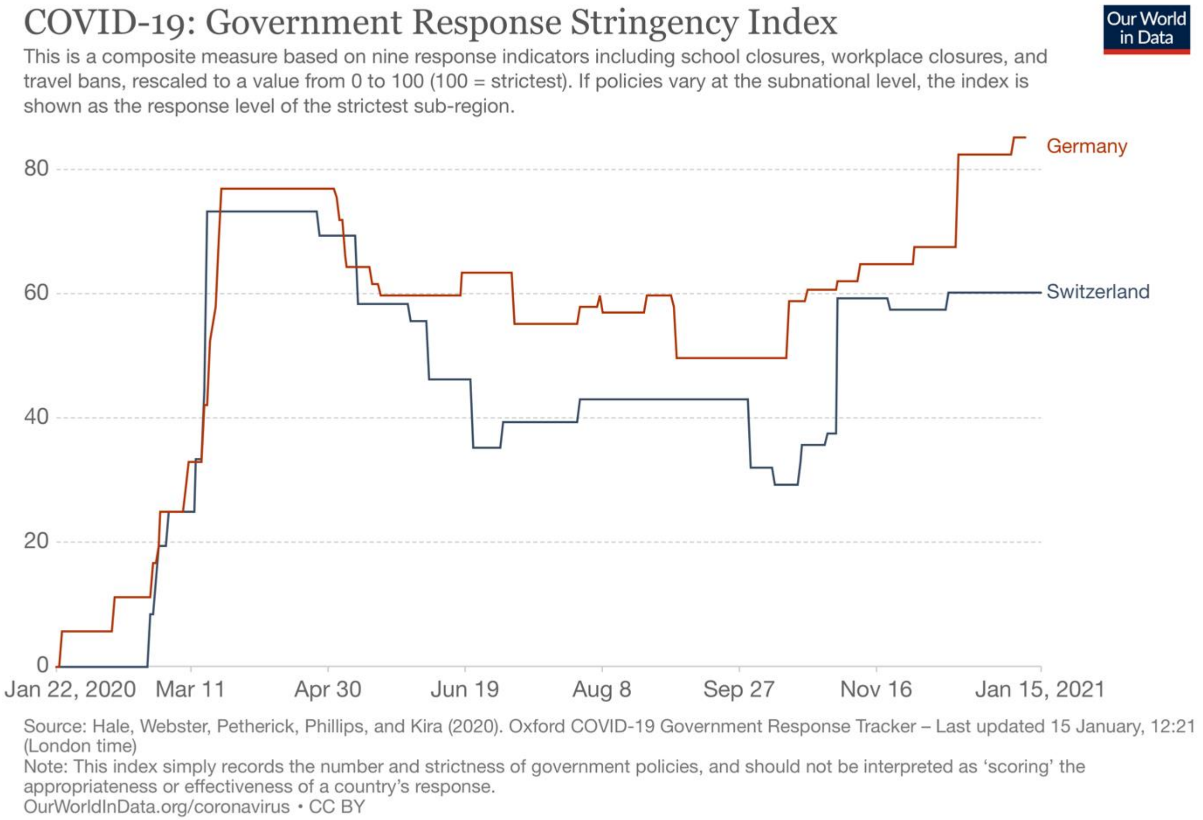

Source: https://ourworldindata.org/grapher/covid-stringency-index
